# P84 Shedding of AMR *Klebsiella pneumoniae* in antibiotic-free poultry and bovine production systems

**DOI:** 10.1093/jacamr/dlaf230.091

**Published:** 2025-12-04

**Authors:** Mian Khaqan Shah, Nicole Kua, Carola Venturini, Sonia Liu, Ruth N Zadoks

**Affiliations:** Sydney School of Veterinary Science, Faculty of Science, University of Sydney, NSW, Australia; Poultry Research Foundation, Faculty of Science, University of Sydney, NSW, Australia; Sydney School of Veterinary Science, Faculty of Science, University of Sydney, NSW, Australia; Sydney School of Veterinary Science, Faculty of Science, University of Sydney, NSW, Australia; Sydney Infectious Diseases Institute, Faculty of Medicine and Health, University of Sydney, NSW, Australia; Poultry Research Foundation, Faculty of Science, University of Sydney, NSW, Australia; School of Life and Environmental Sciences, Faculty of Science, University of Sydney, NSW, Australia; Sydney School of Veterinary Science, Faculty of Science, University of Sydney, NSW, Australia; Poultry Research Foundation, Faculty of Science, University of Sydney, NSW, Australia; Sydney Infectious Diseases Institute, Faculty of Medicine and Health, University of Sydney, NSW, Australia

## Abstract

**Background:**

Zoonotic pathogens pose a serious threat to public health, and are often acquired from foods of animal origin, including livestock and poultry, where they may occur as pathogens or commensals. For example, *Klebsiella pneumoniae*, one of the ESKAPEE organisms, occurs in the gastrointestinal tract of cattle and poultry, and causes mastitis in dairy cows, creating potential routes of transmission via contaminated meat or milk. The occurrence of antimicrobial resistance (AMR) and MDR in ESKAPEE pathogens from animals in high antimicrobial use environments is well-known but there is a limited information available on the occurrence of AMR *K. pneumoniae* in livestock and poultry production systems with restricted antimicrobial use, such as in Australia, which has long limited the use of highest priority critically important antimicrobials in livestock and has very limited use of in-feed antimicrobials.

**Objective:**

This study aimed to investigate the phenotypic and genomic AMR profiles of bovine and poultry *K. pneumoniae*, which may contribute to the spread of AMR to human and other animals via direct contact, contaminated food and environment, even in the absence of dietary antimicrobial growth promoters.

**Methods:**

Bovine *Klebsiella* isolates were obtained from milk of cows with clinical mastitis, using routine bacteriological culture methods and interpretation criteria. Samples were obtained from 2022 to 2024 and originated from 7 herds in New South Wales and Victoria. Only isolates obtained in pure culture, indicative of intramammary infection, and with 500 colony forming units/ml were included. Poultry *Klebsiella* isolates originated from cloacal swabs from broiler chickens. Swabs were collected post-mortem at the end of antimicrobial-free nutrition trials and isolates were obtained from the mixed bacterial population present in such samples using selective agars with and without breakpoint concentrations of antimicrobial compounds. MALDI ToF MS was used for species confirmation of isolates from both host species. Antimicrobial susceptibility testing (AST) was conducted using the disc diffusion method for three antimicrobial agents each of human and veterinary importance (tetracycline, ciprofloxacin, cefotaxime, gentamicin, meropenem and trimethoprim/sulfamethoxazole). Genome sequencing was conducted by Macrogen using the Nextera DNA XT kit for library preparation and NovaseqX (150 bp paired end reads) for sequencing.

**Results:**

AST showed that among all *K. pneumoniae* isolates from poultry (90, obtained from media with and without antimicrobials), 29% showed AMR and 64% showed MDR. Among the MDR isolates, the most common MDR phenotype was observed at 58% to five different antimicrobial compounds having the same MDR profile i.e. tetracycline, ciprofloxacin, cefotaxime, gentamicin and trimethoprim/sulfamethoxazole. While almost all isolates from cattle (52 of 53) were susceptible to all antimicrobial compounds, and none of the bovine isolates were MDR.

**Conclusions:**

Our results show the absence of AMR from bovine *Klebsiella*, despite the use of antimicrobials for control of mastitis on dairy farms, and the presence of AMR and MDR *K. pneumoniae* in poultry in the absence of antimicrobial selection pressure. Thus, factors other than antimicrobial use need to be considered as possible drivers of AMR prevalence and transmission.

